# ORMIR-MIDS: an open standard for curating and sharing musculoskeletal imaging data

**DOI:** 10.1093/jbmrpl/ziag013

**Published:** 2026-01-28

**Authors:** Francesco Santini, Maria Monzon, Simone Poncioni, Serena Bonaretti, Jukka Hirvasniemi, Martijn Froeling, Donnie Cameron

**Affiliations:** Basel Muscle MRI, Department of Biomedical Engineering, University of Basel, 4123 Allschwil, Switzerland; Department of Radiology, University Hospital of Basel, 4031 Basel, Switzerland; Department of Health Science and Technology (D-HEST), ETH Zurich, 8092 Zurich, Switzerland; Swiss Institute of Bioinformatics (SIB), 1015 Lausanne, Switzerland; ARTORG Center for Biomedical Engineering Research, University of Bern, 3010 Bern, Switzerland; Independent Researcher, 8000 Zurich, Switzerland; Department of Radiology and Nuclear Medicine, Erasmus MC University Medical Center Rotterdam, 3000 CA Rotterdam, The Netherlands; Department of Biomechanical Engineering, Delft University of Technology, 2628 CD Delft, The Netherlands; Precision Imaging Group, Center for Image Sciences, Division of Imaging and Oncology, University Medical Center Utrecht, 3584 CX Utrecht, The Netherlands; Department of Medical Imaging, Radboud University Medical Center, 6525 GA Nijmegen, The Netherlands

**Keywords:** CT, data curation, digital radiography, medical image processing, MRI, musculoskeletal system, open source software, standards

## Abstract

Quantitative imaging is increasingly used for both clinical and research applications in musculoskeletal (MSK) disorders. Its widespread use, coupled with an assortment of modalities and vendors, has led to a diverse range of analysis methods and challenges in reproducing results both within and across centers. Clearly, consensus is needed to establish consistent data organization principles and thus permit the use of standardized image processing pipelines. Here, we—members of the Open and Reproducible Musculoskeletal Imaging Research (ORMIR) community—introduce the ORMIR-MIDS data format, which is derived from the existing Brain and Medical Imaging Data Structures (BIDS and MIDS) and extends them to MSK applications. ORMIR-MIDS comprises both a standard specification and a set of software tools for data conversion and organization. The latter permits the conversion of image data to a standardized format, in an organized folder structure, and produces up to 3 metadata files: important data-processing information, patient data, and complete image header tags to allow conversion back to the original format. The tool currently supports a range of imaging modalities and sub-modalities relevant to the MSK system, with more to come in the future. A suite of test data is also provided to demonstrate the functionality of the software, and the file system structure and associated image files and metadata after conversion of these test data are demonstrated here. With ORMIR-MIDS, we provide an open specification for multimodal MSK imaging, alongside tools for creating compliant datasets. Adherence to our standard will improve harmonization of imaging data across vendors and institutions and permit the development of reproducible processing pipelines and data repositories for MSK research.

## Introduction

Imaging in general, and quantitative imaging in particular, is becoming an essential tool for evaluating musculoskeletal (MSK) conditions. At the same time, current trends in research are favoring data-driven methods such as machine learning for the development of novel diagnostic tools and outcome measures. This situation results in large quantities of data to be produced, stored, and transmitted, which then need to be aggregated and processed jointly. Alongside these practical requirements, rising interest in open and reproducible research, both from research institutions and funders,^1–3^ has encouraged the sharing of data such that they are Findable, Accessible, Interoperable, and Reusable (the “FAIR” principles^4^). In this context, an evolution of the data formats used for storing imaging data for research purposes has been inevitable.

Various imaging modalities use a number of imaging formats. Some of them are proprietary, others, such as the widely-used “Digital Imaging and Communications in Medicine” (DICOM)^5^ format, offer a level of standardization and interoperability, such that images can be transmitted, stored, and viewed by common tools used in hospitals and clinical practices. However, this level is sometimes insufficient for proper data aggregation, as different vendors (and sometimes different software versions from the same vendor) adopt different conventions, within the standard, for storing metadata information. The guidelines are open to different interpretations, so that, for instance, different metadata fields can be used to store the same or similar information (eg, the DICOM fields “Scanning Sequence” and “Echo Pulse Sequence”), or private, nonstandardized fields can be added to the file at the discretion of the creator.

Several initiatives have been trying to improve harmonization, especially in the field of brain imaging. The best known, and probably most successful, has been the Brain Imaging Data Structure (BIDS)^6^ format. This format standardizes 4 aspects of the data:

- the folder structure;
- the format in which the “raw” image data are stored, in this case the Neuroimaging Informatics Technology Initiative (NIfTI) format^7^;
- the format of the metadata associated with each image/3-dimensional volume, provided in JavaScript Object Notation (JSON), and which metadata fields are required for each acquisition;
- the format of the patient-relevant data (tab-separated values).

This format has been developed for brain imaging, and, originally, for functional MRI (fMRI), so its associated prescriptions are specifically tailored for this usage. Over the years, extensions and modifications to the BIDS format have been proposed. For example, BIDS for quantitative MR,^8^ BIDS for MR spectroscopy,^9^ BIDS for Near-Infrared Spectroscopy,^10^ and the Medical Imaging Data Structure (MIDS),^11^ which aims to include multiple modalities and body regions. So far, no such standard exists for MSK imaging, which comprises multiple organs (muscular tissue, bones, cartilage, and tendons) and multiple routinely-used modalities (CT, conventional radiography, ultrasound, and MRI). Further, some MSK pathologies, like neuromuscular or rheumatic disorders, have low prevalence, and the aggregation of data from multiple sources is crucial to achieve reliable results, requiring multicentric studies to evaluate the efficacy of novel therapies.

We—the Open and Reproducible Musculoskeletal Imaging Research (ORMIR) community^12^—are developing a new data format, termed ORMIR-MIDS, derived from and extending the MIDS format, which unifies the needs of the broad MSK imaging research community across multiple modalities, acquisition types, and regions of interest. Our approach broadly aligns with the existing BIDS and MIDS standards, deviating only where beneficial to MSK applications. To make our standard usable and accessible, we are also developing software tools to both automatically convert proprietary or semi-proprietary data formats into a format conforming to our standard, and to incorporate this format into custom tools, to remove the burden of correctly supporting a plethora of input and output conventions, with the goal of making them fully interoperable.

## Materials and methods

### The ORMIR-MIDS standard

ORMIR-MIDS uses the NIfTI file format for image data and the JavaScript Object Notation (JSON) for metadata. It extends BIDS by adding clinical and research imaging modalities—such as (quantitative) CT and plain radiography—that are widely used in the MSK imaging community, along with the essential metadata for image processing. Like BIDS, our new standard organizes data into specific folder structures with naming conventions to assist image identification and access.

**Figure 1:**
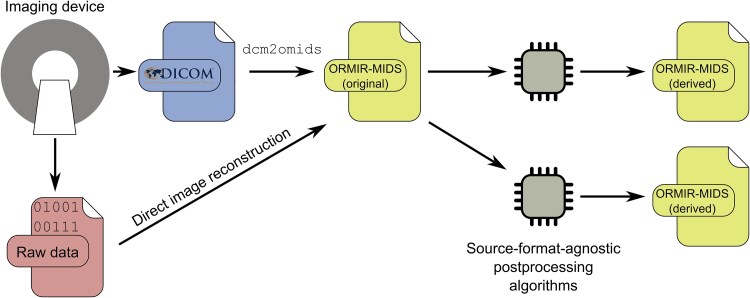
Pipeline using ORMIR-MIDS as a generic file format. ORMIR-MIDS data can be obtained from DICOM images through the bundled converter, or can be directly obtained from the imaging device raw data by direct image reconstruction. In any case, this allows subsequent post-processing steps, applied by the user, to be agnostic to the source format of the images. Newly-generated images, derived from these post-processing steps, can also be saved to the ORMIR-MIDS format.

ORMIR-MIDS is intended to be a generic, source-agnostic format to be used both as input and output for postprocessing algorithms, to avoid supporting multiple proprietary conventions (see Figure 1).

The ORMIR-MIDS standard is the extension and continuation of the preceding muscle-bids package (https://muscle-bids.github.io/), which was originally defined through consensus among volunteers from the muscle MR imaging community (indicated on the main page of the muscle-bids project). The muscle-bids project was then adopted by the ORMIR community during the in-person workshop “Sharing and Curating Open Data in Musculoskeletal Imaging Research,” which took place in Zurich (Switzerland) in January 2024. The standard was extended to other modalities based on the experience of the multidisciplinary team that was present at the workshop, and continues to be developed by volunteers of the community through a consensus-seeking process and regular meetings every 6 weeks.

#### File system structure

The output file system structure for ORMIR-MIDS comprises a participant-specific root folder containing subfolders describing the kind of data contained therein (eg, CT, MRI anatomical). These data consist of one NIfTI image file, with a filename corresponding to the participant and a filename suffix representing the acquisition, and up to 3 standardized JSON header files:

- A minimal BIDS-like header with parameters required for interpreting image data;
- A new, optional header with sensitive patient information; and
- Another new, optional header containing all original DICOM image header tags.

Inclusion of patient information permits simple anonymization, whereas the saved DICOM tags permit bidirectional conversion between ORMIR-MIDS and other image formats, which is important for interoperability, for standardized data sharing, and for uploading processed data to picture archiving and communication systems.

To facilitate statistical comparisons of imaging data and participant demographic and health data, ORMIR-MIDS also adds further participant health-related data to the standard BIDS participant description table (“participants.tsv”), with the option to include raw clinical data in participant sub-folders.

Following the conventions of MIDS, ORMIR-MIDS currently defines a folder structure able to accommodate multiple modalities and MR acquisition types. This also allows users to save their own derived images—such as quantitative maps generated via their own postprocessing algorithms—to the same structure. It is good practice, however, to keep such derived data separate from the source data. Ideally, derived data should be stored in a “derivatives” subfolder comprising the same subject and session structure as the source data, as recommended by the BIDS standard^13^; however, this is not currently mandated in the ORMIR-MIDS format.

The standard does not currently support raw data storage, only reconstructed data; however, other standards exist for MRI raw data, such as the International Society for Magnetic Resonance in Medicine Raw Data (ISMRMRD) format.^14^ The current full ORMIR-MIDS specification is available at https://ormir-mids.github.io/specs. The specification indicates required JSON fields, which provide the necessary metadata for the interpretation and reproduction of the acquisition.

#### Supported modalities

The inclusion of imaging (sub-)modalities in our standard is subject to a number of considerations. Many have one or more potential acquisition or reconstruction formats, and dedicated header tags that are required for their interpretation, which are—in most cases—not included in the original BIDS specification. Table 1 summarizes all imaging submodalities currently included, or planned to be included, in ORMIR-MIDS. An up-to-date list is provided on the website (https://ormir-mids.github.io/specs), along with a roadmap of planned additions (https://github.com/orgs/ormir-mids/projects/2). Further details are provided in the remainder of this section.

**Table 1:** Imaging (sub-)modalities included, or planned for inclusion, in ORMIR-MIDS.

**Status**	**Modality**	**Folder** **name(s)**	**File name** **suffix**	**Image** **volume**	**ORMIR-MIDS JSON fields**
**Included**	Computed tomography	ct	ct, pcct, hrpqct	3D (x, y, z)	XRayEnergy [kVp] XRayExposure [mAs] ConvolutionKernel RescaleIntercept RescaleSlope ScancoMuScaling ScancoDensitySlope ScancoDensityIntercept ScancoMuWater
	Computed/plain radiography	cr	N/A	2D (x, y)	ExposureTime [ms] X-RayTubeCurrent [mA]
	MRI: Quantitative T1/T2/wT2	mr-quant	t1/t2/wt2	3D (x, y, z)	
	MRI: DESS	mr-anat	dess, dess-fid, dess_echo	3D	PulseSequenceType: “DESS”
	MRI: Multi-echo gradient echo	mr-anat	megre	4D (x, y, z, echo)	EchoTime (array) in ms WaterFatShift in pixels MagneticFieldStrength
	MRI: Multi-echo spin echo	mr-anat	mese	4D (x, y, z, echo)	EchoTime (array) in ms RefocusingFlipAngle in degrees
**In progress/Planned**	MRI: Velocity quantification	mr-quant	vel	5D (x, y, z, t, direction)	FourthDimension: TriggerTime (array) in ms FifthDimension: VelocityEncodingDirection [array, vectors] Venc (array) in m/s
	MRI: Diffusion	mr-quant	diff	4D (x, y, z, direction)	FourthDimension: EncodingDirection [array, vectors] MixingTime in ms
	Segmentation	seg	seg	3D (x, y, z) 4D (x, y, z, label)	Labels (array of strings)
	Ultrasound	us	t.b.d	3D (x, y, z/time)	t.b.d.

Abbreviations: DESS, double echo steady state; FS, fat-suppressed; JSON, JavaScript Object Notation; ORMIR-MIDS, Open and Reproducible Musculoskeletal Imaging Research Medical Imaging Data Structure; T1w, T1-weighted; T2w, T2-weighted; t.b.d., to be determined; wT2, water T2.

#### Magnetic resonance imaging

##### Multi-echo spin-echo (MESE) MRI

This sequence is widely used for T2_water_ determination in skeletal muscle,^15–17^ or for T2 relaxometry in cartilage.^18^ Complex data (magnitude/phase) can be included per echo.

##### Multi-echo gradient-recalled-echo (MEGRE) MRI

This approach is used primarily for chemical-shift-based water-fat-separation, or “Dixon” imaging, for estimation of muscle fat content. Methods range from a semi-quantitative 2-point Dixon approach^19^ to multi-point quantitative approaches.^20,21^ Data can include complex images (magnitude/phase or real/imaginary) per echo, which can also serve as in- or opposed-phase images, whereas derived images can include fat- or water-fraction maps, B0 maps, T2^*^ or R2^*^ maps, or calculated in- or opposed-phase images. Other examples of MEGRE MRI include T1 and T2 mapping or MR fingerprinting, which are increasingly used in MSK imaging research.^22–24^

##### Double-echo MRI

A special case of MEGRE MRI, known generically as FAst Double Echo (FADE) or variously as Double Echo Steady State (DESS) or Multi-Echo iN Steady-state Acquisition (MENSA) by vendors.^25,26^ This method is predominantly used for assessment of knee cartilage. Here, both a free induction decay and gradient echo are acquired and are either reconstructed separately or combined into a single image or fitted T2 map.

#### Computed tomography

##### Energy-integrating-detector CT (EID-CT)

Conventional CT with energy-integrating-detector (EID) technology is widely used in MSK imaging and research. It provides information, for example, on skeletal anatomy and MSK pathology. Data from image reconstructions can be included.

##### Photon-counting CT (PC-CT)

This emerging imaging modality provides improved spatial resolution and dose efficiency compared to the conventional EID-CT. Photon-counting detectors improve image quality and reduce noise, which enables improved visualization of fine anatomical structures, such as trabecular bone, subchondral cysts, and osteophytes.^27^ Data from different image reconstructions can be included.

##### High-resolution peripheral quantitative CT (HR-pQCT)

This 3D imaging technology has the capacity to scan peripheral sites, such as the distal radius and tibia. The second generation of scanner (XtremeCT II, Scanco Medical) provides a resolution of 90 μm at 10% modulation transfer function, which allows for the distinction between cortical and trabecular compartments. It exhibits a higher sensitivity to microstructural changes with respect to DXA, while maintaining a low effective radiation dose of 3-5 μSv per clinical stack (10.2 mm in thickness along the metaphysis). Further, the integration of advanced computational methodologies within the scanner enables rapid assessment of bone structural mechanics using the finite element method. This imaging modality is mainly used to assess bone microarchitecture and its longitudinal variations. Its applications include understanding the pathophysiology of interventions for osteoporosis treatments, bone health diseases, and fracture healing.^28^

##### Plain radiography

Plain radiography is the most widely used modality in MSK imaging and a standard assessment tool in MSK research. It provides projectional images of bone, joint alignment, and gross soft-tissue changes.

### The ORMIR-MIDS Python package

The Python package offers tools for: (1) converting DICOM datasets to the ORMIR-MIDS format and (2) loading existing ORMIR-MIDS datasets for subsequent processing by automatically loading the NIfTI files and the JSON metadata. Using an approach based on Pyvoxel (https://github.com/pyvoxel/pyvoxel), these data are made accessible as a data structure compatible with NumPy. The package can be installed from the Python Package Index via pip.

#### Image converters

Central to the ORMIR-MIDS package is an automated tool for the conversion of selected DICOM image series into ORMIR-MIDS format, based on a plugin-like hierarchical structure of classes called “Converters.” These recognize the acquisition type from the list given in the “Supported Modalities” section, and automatically populate the required JSON fields, accounting for different vendor conventions as needed. For MRI data, our image converters diverge from the BIDS standard by saving multi-echo data as 4-dimensional NIfTI files—one per complex image type. The BIDS standard uses the fourth dimension to store time series, in the case of fMRI or arterial spin labeling, or diffusion encoding in the case of diffusion MRI. For MSK imaging, using the fourth dimension to store multi-echo data simplifies file-handling for MESE and MEGRE, where large numbers of echoes can be acquired. For complex data, although the NIfTI file format supports 32- and 64-bit floating complex pairs, we maintain the BIDS convention of separating magnitude and phase (“part-phase”), or real and imaginary (“part-real” and “part-imag”), data into separate volumes.

#### Test datasets

Representative imaging data have been provided in a Zenodo repository,^29^ and currently include MEGRE and MESE MRI data collected on GE HealthCare and Philips Healthcare systems, respectively. Representative imaging data for HR-pQCT systems are provided in a separate Zenodo repository.^30^ Further test data will ultimately be included for all supported modalities and vendors. The Python package also includes unit and integration tests to verify the download and conversion of the test data, as well as the structure of the output NIfTI files and the contents of the JSON sidecar files.

A Jupyter notebook has been developed to showcase the current functionality of ORMIR-MIDS: https://ormir-mids.github.io/tutorials.

## Results

### Test data: output structure

**Figure 2:**
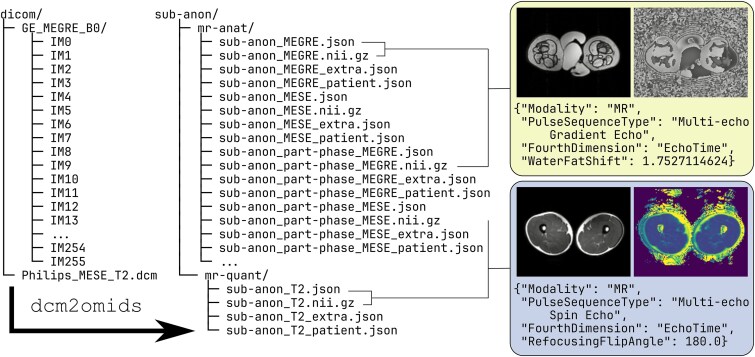
Illustration of DICOM-to-NIfTI conversion with ORMIR-MIDS, including the output file system structure. As is apparent from the image, the converter produces the imaging volume in compressed NIfTI format, along with the JSON headers, but leaves the task of creating the “sessions” level of the folder tree to the user. Abbreviations: MEGRE, multi-echo gradient-recalled echo; MESE, multi-echo spin-echo.

Figure 2 illustrates a condensed version of DICOM-to-NIfTI conversion via the ORMIR-MIDS image converters and shows the output structure and example images and metadata from one of our test datasets. The conversion process shown here can be executed either as a Python function or via the command line.

In brief, the test data comprise both individual “classic” DICOM MEGRE files, containing GE HealthCare MEGRE data, and a single multi-frame “enhanced” DICOM file, containing Philips Healthcare MESE data. Image converters are tested in series for consistency with the loaded DICOM image header tags, based on modality, manufacturer, and MRI scanning sequence. Acquired and derived images are then converted to NIfTI based on the “image type” parameter, and essential header tags are extracted, such as pixel bandwidth for MEGRE and refocusing flip angle for MESE. The latter are saved in JSON sidecar files alongside the NIfTI data, along with an “extra” JSON file containing the full DICOM header and, optionally, a “patient” JSON file containing patient information. The output data are arranged as described in the “File system structure”—being organized into directories per modality, with MRI data being split into anatomical (“mr-anat”) and quantitative (“mr-quant”) data directories for harmonization with the BIDS standard.

### Real-world examples

Following are 2 examples in which ORMIR-MIDS is already used as the preferred input/output format for reconstruction and postprocessing pipelines.

#### MyoQMRI 2.0

This package (https://github.com/BAMMri/MyoQMRI) contains tools for the acquisition, reconstruction, and analysis of quantitative MR images of the skeletal muscle. It contains a vendor-independent implementation of an MESE sequence (developed using PyPulseq^31,32^), and scripts for: the reconstruction of the acquired images, the fitting of T2_water_ through extended-phase-graph simulation,^16^ and the calculation of fat/water separated images from MEGRE images. ORMIR-MIDS is used as the output format of the reconstruction script of the MESE raw *k*-space data, and also as input and output of the T2_water_ and fat/water fitting scripts. Output maps are compatible with the specifications and stored in the “mr-quant” subdirectory of the dataset. The T2_water_ fitting script also accepts DICOM images as input, but uses the ORMIR-MIDS package to load them and convert internally to the ORMIR-MIDS object structure.

#### Open-source, accelerated velocity quantification MR sequence

This software package (https://github.com/BAMMri/Pulseq-4DFlow) contains a PyPulseq implementation of a 3-dimensional, 3-directional phase-contrast gradient echo sequence for velocity quantification^33^ with undersampled acquisition, and a reconstruction pipeline based on the ISMRMRD format^14^ as input, and ORMIR-MIDS as output.

## Discussion

In this paper, we have described the ORMIR-MIDS standard, which is a proposed ad hoc format for the storage and dissemination of medical images for MSK applications, encompassing multiple imaging modalities.

Currently, the definition of the standard comprises MRI, CT, and plain radiography, including multiple submodalities and contrasts within each modality, with the plan to expand to ultrasound and, potentially, optical imaging and magnetic resonance spectroscopy in the future. The standard stems from the BIDS standard and borrows many aspects from it; however, BIDS maintains an MR- and brain-centric approach, by defining an “anatomy” modality folder as containing MR images by default. ORMIR-MIDS instead adopts a multi-modality, multi-organ approach, similar to that proposed by the more-general MIDS definition.^11^ MIDS adds an additional directory layer to BIDS, including modality folders for ultrasound and nuclear medicine, for example, and nesting the BIDS “anat” and “quant” folders inside an MRI modality folder. The ORMIR-MIDS specification, on the other hand, envisages “mr-anat” and “mr-quant” as MR-folders at the same directory level as “ct” (for CT) and “cr” (for conventional radiography). The inclusion of additional modalities is partly a matter of definitions, however, as BIDS has already been extended to support multiple modalities, including microscopy,^29^ positron emission tomography,^30^ and more. In terms of metadata, ORMIR-MIDS maintains most of the default common JSON fields prescribed by the BIDS standard, while adding application-specific fields that are relevant for the specific workflows of MSK imaging. An example is the inclusion of the excitation and refocusing slice profiles in the multi-echo spin-echo acquisition. These are relevant for the correct calculation of water T2 using extended-phase-graph fitting methods, which are currently the reference standard in muscle imaging.^15–17^ Also, in contrast to the BIDS standard, the ORMIR-MIDS specification was developed with anonymity in mind, creating an additional JSON header that contains potential patient-identifying information, and which can be easily removed from the dataset. Fields, such as the “institution name,” which are included in the standard BIDS header, are moved to this patient-specific header in ORMIR-MIDS. Lastly, ORMIR-MIDS simplifies file-handling relative to BIDS by collating multi-echo MRI data into a single 4-dimensional NIfTI file, as opposed to multiple, individual image volumes. Crucially—despite the aforementioned differences, and slight changes in naming conventions—ORMIR-MIDS is broadly compatible with both BIDS and MIDS. This will simplify interoperability with existing software tools that interact with either of these standards, and will permit data from BIDS, MIDS, or ORMIR-MIDS to co-exist in the same structure.

Together with the specification, we provide an ad hoc converter that aims to convert DICOM data into ORMIR-MIDS-compliant data. This is not the only existing software tool with such an aim, the most popular one being the dcm2niix package.^34^ This latter tool aims to be a generic converter for DICOM images, extracting BIDS-compliant information from the DICOM header in order to produce BIDS-like files from the widest range of source acquisitions. The ORMIR-MIDS converter, in contrast, has the opposite approach: it aims to identify specific acquisitions that are compliant with the ORMIR-MIDS specifications and only convert the datasets that are explicitly included, resulting in a minimal set of outputs that are strictly compliant with the standard, both in terms of acquisition types and metadata included in the JSON header. This ensures anonymity, by excluding fields that are not explicitly included in the specifications, and that might contain patient-identifying information (eg, image comments), and strict compliance, as the final JSON header is guaranteed to include all and only the fields required by the standard. This approach of only converting the minimum set of strictly compliant data has the main limitation of excluding potentially compatible datasets unless explicitly identified in the converter code. To overcome this limitation, the converter tool is developed with a modular architecture, through which new datasets can be supported by creating converter modules that follow a common application programming interface.

In the real-world examples presented above, ORMIR-MIDS enables the application of each part of the pipeline independently from the origin of the data. When coupled with other vendor-agnostic tools, such as the sequence-development framework PyPulseq,^32^ which is compatible with all the major MR scanner vendors, and the ISMRMRD standardized MR raw data format, ORMIR-MIDS enables full pipelines (from acquisition to postprocessing) to be realized without dealing with vendor-specific implementations.

There are still many aspects of MSK imaging that are not yet covered by ORMIR-MIDS, the aforementioned ultrasound being one. Other examples that are still in progress, both in terms of definition and conversion, are MR diffusion imaging and MR velocity mapping. To develop these aspects, ORMIR-MIDS is being treated as an open standard, to which interested parties working on specific modalities are encouraged to contribute, either through direct communication with the ORMIR community or via GitHub. The “Contributing new specifications” page of the ORMIR-MIDS website (https://ormir-mids.github.io/new_spec) defines the guidelines for community contribution of novel specifications. Users inside and outside the ORMIR community are encouraged to create a “pull request” following a standardized template, in which they define the proposed file naming convention, the dimensionality of the data for the proposed acquisition, and the required JSON fields. New proposals are reviewed by the ORMIR-MIDS working group, which currently meets regularly every 6 weeks to discuss such extensions and development directions. As with every open-source software package developed within academia, continued maintenance of the software is a concern. While it is no absolute guarantee of longevity, ORMIR-MIDS is supported by a multidisciplinary and international growing community currently counting more than 60 members at all levels of academic ages, which can provide continuity in the development and update process. As additional sources of continuity, we plan to explore collaborations with existing initiatives, mostly active in the field of neuroimaging, such as the BIDS project itself, and the recently launched European Cooperation in Science and Technology (COST) action on neuroimaging data sharing (Improving Neuroimaging Data for Sharing (INDoS), https://www.cost.eu/actions/CA24161/).

In conclusion, ORMIR-MIDS aims to provide both an open specification for multimodal MSK imaging, and practical tools to convert and create compliant datasets. It is a community-driven project that is expected to receive long-term support and input from across disciplines within the MSK field. Ultimately, adherence to our ORMIR-MIDS standard will improve harmonization across vendors and institutions, and improve interoperability of tools to create reproducible processing pipelines and data repositories for MSK research.

## Data and code availability statement

The version of the ORMIR-MIDS specification website at the time of submission is accessible at the following immutable URL: https://doi.org/10.5281/zenodo.17980944. The version of the ORMIR-MIDS Python package at the time of submission (v0.1.3) is accessible at the following immutable URL: https://doi.org/10.5281/zenodo.17980939
